# Shaping Ability of XP Endo Shaper File in Curved Root Canal Models

**DOI:** 10.1155/2020/4687045

**Published:** 2020-02-17

**Authors:** Abdulmohsen Alfadley, Abdalrhman Alrajhi, Hamad Alissa, Faisal Alzeghaibi, Lubna Hamadah, Khalid Alfouzan, Ahmed Jamleh

**Affiliations:** ^1^Restorative and Prosthetic Dental Sciences, College of Dentistry, King Saud Bin Abdulaziz University for Health Sciences, National Guard Health Affairs, Riyadh, Saudi Arabia; ^2^King Abdullah International Medical Research Centre, National Guard Health Affairs, Riyadh, Saudi Arabia

## Abstract

The aim of this study was to assess the shaping ability of the XP Shaper (XPS) file in severely curved canal models under simulated body temperature and compare it with that of the WaveOne Gold (WOG) file. Ninety-six simulated root canals were equally distributed into XPS and WOG systems to be shaped by eight files each. Files were assessed under a stereomicroscope prior to canal shaping to detect deformation if any. The canals were shaped at 35 ± 1°C using the X-Smart Plus motor. Images of the canals were obtained before and after instrumentation using a stereomicroscope to measure the amount of removed resin from both the inner and outer curvature sides at apex (0 mm) and 3 mm and 6 mm from the apex. The shaping time was calculated. The data were statistically analyzed by the independent *t*-test at 5% significance level. The XPS and WOG systems shaped the canals in 37.0 ± 9.5 and 62.6 ± 11.3 seconds (*P* < 0.05), respectively. At the apex level, the amount of resin removal in both sides did not show a significant difference between the tested groups (*P* > 0.05). At 3 mm and 6 mm levels, the WOG removed more resin than XPS at both sides (*P* < 0.05). In XPS, deformation was observed in four files: one file after the first use, one file after the fourth use, and two files after the sixth use. In WOG, two files were deformed: one file after the fifth use and one file after the sixth use. One XPS file was fractured after the sixth use. In short, XPS and WOG files can be used in shaping severely curved canals as they showed the ability to maintain the original shape with minimal transportation. Both file systems showed signs of deformation after use with a lower number of deformed files observed in WOG throughout the experiment.

## 1. Introduction

High-quality chemomechanical debridement of the intricate root canal system is paramount to the success of endodontic therapy. This is required in order to eradicate bacteria and its metabolic substrate which are responsible for the initiation and persistence of endodontic disease [1]. The endodontic file is used to remove intracanal pulpal tissues, microbial biofilm, and toxic byproducts and to develop a continuously tapering canal while maintaining the canal geometry that ultimately allows for the delivery of irrigating solutions and intracanal medicaments as well as the three-dimensional filling of the root canal system [2]. Historically, stainless steel hand files have been used to perform canal shaping. However, these files are stiff and associated with increased operator fatigue, and when used in the preparation of curved root canals, the restoring forces of the files tend to return the file back to its original shape, resulting in canal transportation [3]. This issue is further complicated by the fact that most of the root canals are curved, wherein preserving the original canal anatomy is critical for attaining favorable treatment outcomes [4]. Nowadays, nickel-titanium (NiTi) files are broadly used to shape the root canals owing to their increased flexibility, rapid and centered canal preparation, safer preparation of curved canals, improved cutting efficiency, and improved treatment outcome [3, 5]. In spite of all these advantages, the main limitation in the use of a NiTi file is the risk of their fracture, especially when it is autoclaved and reused [6, 7]. The NiTi file fracture may occur from torsional fatigue, cyclic fatigue, or both [8].

Literature showed that canal shaping is influenced by many factors such as file's alloy and geometry, thermomechanical manufacturing, and motion kinematics [9–15]. Contemporary advancements in NiTi file production contributed to the evolvement of single NiTi systems, in which the mechanical preparation of the root canal is completed using one file. The use of single NiTi files is considered beneficial as it reduces the preparation time, cost, and risk of cross-contamination [16]. Furthermore, it was shown that canal shaping with single-file systems did not compromise canal cleanliness [10].

XP-Endo Shaper (XPS; FKG Dentaire SA, La Chaux-de-Fonds, Switzerland) is a single-file system that is used in a continuous rotary movement. This file is snake-shaped with a triangular cross-section. It has an apical diameter of 0.27 mm and a fixed taper of 0.01. The MaxWire technology involved in the production of this file provides it with superelasticity and shape memory properties [17]. Upon exposure to body temperature (35°C), the martensite phase of the file converts to the austenite phase, and the taper increases to 0.04 according to the molecular memory of the A phase [18]. The file presents a six-blade tip, the Booster tip, that allows it to start shaping the canal after a manual glide path of at least size 15 ISO and to gradually increase the apical size to achieve an ISO size 30. XPS achieves a final apical preparation of at least 30/0.04 [17–19]. XPS was found to have superior cyclic fatigue performance compared to other file systems [19–22].

WaveOne Gold (WOG; Dentsply Sirona, York, PA) system is an updated version of the WaveOne (WO; Dentsply Sirona) single-file system. WOG file is operated in reciprocating motion, has a noncutting tip, and exhibits a variable taper design. WOG file is made up of “gold wire” wherein the manufacturing process involves postmachining heat treatment of the files followed by a cooling cycle [12]. Moreover, the WOG file presents with a unique alternating off-centered parallelogram-shaped cross section, along with 2 cutting edges [12, 13].

Previous studies investigated the shaping ability of XPS in oval-shaped and large canals [23–25] in canals with curvatures of 10–20° [26, 27] and 25–40° [28]. However, no study has addressed its shaping ability in narrow and severely curved (60° angle) canals so far. Thus, the aim of this study was to evaluate the shaping ability of XPS in J-shaped canal models and compare it with that of WOG. The null hypothesis stated is that there is no difference between XPS and WOG files in terms of shaping ability.

## 2. Materials and Methods

### 2.1. Sample Preparation

Ninety-six simulated resin canal blocks with a J-shaped canal (Endo training bloc, 2% taper, 17 mm length; Dentsply Sirona, York, PA) were randomly divided into two experimental groups (*n* = 48 each) according to the preparation systems, XPS and WOG.

### 2.2. Canal Shaping

The working length (WL) was set on the canal exit of the resin canal blocks. A glide path was established with a #15 K-file to the WL. The XPS file was operated at 1000 rpm speed and 1 N·cm torque. The file was inserted in the canal, and 5 strokes were applied (in-and-out motion) until the file reached 0.5 mm shorter of the WL (adjusted WL) as recommended by the manufacturer. To ascertain the completion of canal shaping, a gutta-percha cone size 30, 0.04 taper was fitted to within 0.5 mm of the WL. In WOG, canals were shaped by inserting the WOG primary instrument three times in a slow in-and-out pecking motion by using the “WAVEONE ALL” mode in X-Smart Plus (Dentsply Sirona, York, PA). This was repeated until the file reached the WL. Before canal shaping, the resin model was immersed in a warm water bath at 35 ± 1°C up to the canal opening to simulate clinical conditions throughout the experiment. The canal shaping was performed by a previously trained operator in both systems using the X-Smart Plus motor (Dentsply Sirona). After each file insertion, the canal was irrigated with a 1% sodium hypochlorite solution, and canal patency was controlled with a size-10 K-file, and the file flutes were cleaned with wet gauze. After complete shaping, each canal was rinsed with 1 mL 17% EDTA. Shaping time was recorded in seconds by including the time required for canal shaping, canal irrigation, and recapitulation. After each canal shaping, the file was sterilized by using a steam autoclave. Each file was used up to six times. If any deformation/fracture was detected, the file was discarded and the number of file uses was counted.

### 2.3. Assessment of Canal Shaping

Images of each sample were taken before and after shaping by a digital stereomicroscope (Leica EZ4 HD, Leica Microsystems, Singapore). A composite image of the superimposed pre- and post-operative images was produced by using Adobe Photoshop software. The amounts of removed resin from the walls were determined from both inner and outer curvature sides at 0 mm, 3 mm, and 6 mm levels from the canal exit. These measurements were performed by a second operator who was blinded to the experimental groups.

The canal transportation value was calculated according to this formula: the amount of resin removed from the outer side minus the amount of resin removed from the inner side, at the three levels [29]. Based on this formula, a value of 0 indicates perfect centering ability with no canal transportation. Positive and negative values, however, indicate the presence of transportation to the outer and inner sides, respectively.

### 2.4. Assessment of Files

All files were examined under a dental operating microscope (OPMI pico; Carl Zeiss, Gottingen, Germany) at 20X magnification before each use for any possible deformation or fracture.

### 2.5. Analysis of Data

The data of preparation time and amount of root canal wall cutting at three levels in the apical 6 mm were statistically analyzed by the independent *t*-test. The lifespan of the tested files was estimated using survival analysis. A significant difference was detected at a level of *P* < 0.05.

## 3. Results

**Figure 1:**
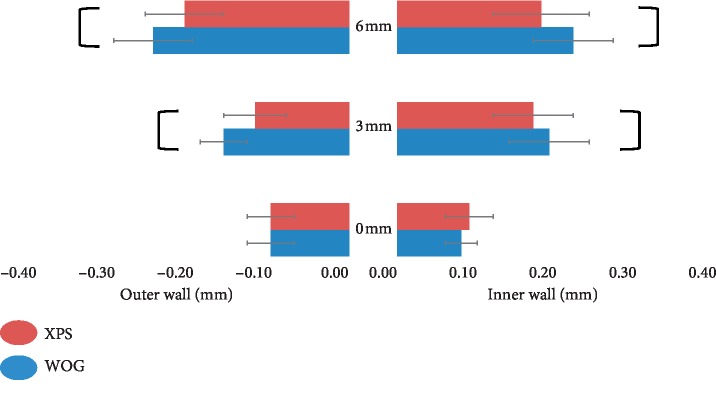
The amount of root canal wall cutting at the apex (0 mm) and 3 mm and 6 mm from the apex. The bars indicate significant differences between the groups.

The XPS and WOG systems were able to instrument the canals in 37 ± 9.46 and 63 ± 11.33 seconds (*P* < 0.05), respectively. The amount of resin removal in the inner and outer sides at the apex (0 mm) did not show significant differences between the tested groups (*P* > 0.05). At 3 and 6 mm from the apex, however, the WOG removed significantly more resin from the inner and outer curves than its XPS counterpart (*P* < 0.05) (Figure 1). In all canals shaped with XPS, a successful fitting of gutta-percha cone size 30, 0.04 taper within 0.5 mm of the WL was obtained.

In terms of canal transportation, there were no significant differences between the experimental groups at the three levels. At 0 mm and 6 mm, the root canals were transported more to the outer side of the curvature after shaping in both groups (*P* > 0.05). At 3 mm level, the canals were transported more to the inner side of the curvature in both groups (*P* > 0.05) (Table 1).

**Figure 2:**
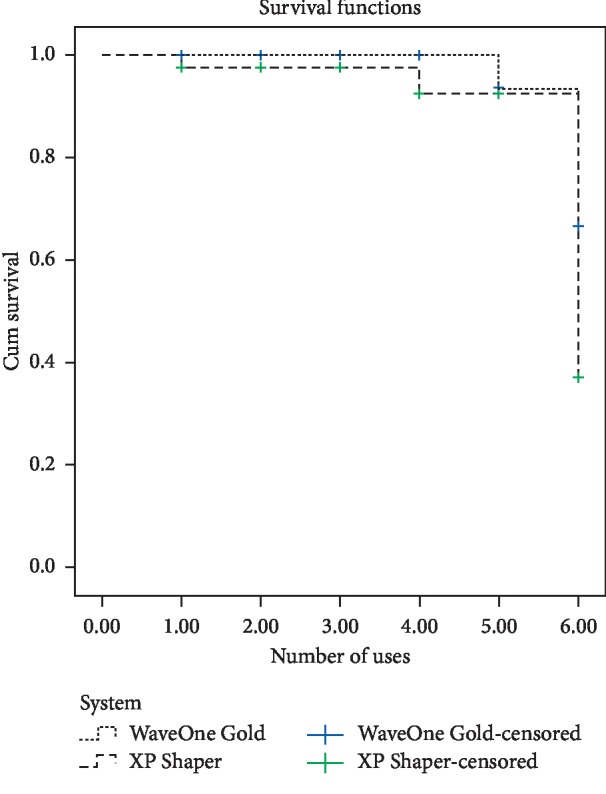
The survival curves for the experimental groups according to the file system.

The survival curves for the two experimental groups are shown in Figure 2. Three WOG files were deformed: one after the fifth use and two after the sixth use. One XPS file fractured in the sixth use, and four XPS files were deformed: one after the first use, one after the second use, and two after the sixth use.

## 4. Discussion

Preserving the original anatomy of the root canals without canal transportation is an important mechanical objective in canal shaping. This in vitro study evaluated the shaping ability of XPS and WOG in narrow and severely curved canals by using J-shaped root canal models with a 10 mm curvature radius and 60° curvature angle. At the canal exit level, the amounts of resin removal in both sides were comparable although the tip sizes and tapers of XPS and WOG files are different. This might be attributed to many factors such as the file's properties, geometry, and the mechanism of action. The XPS file has a 1% taper that, under intracanal temperature, will appear as a snake in shape which allows shaping the canal in three dimensions with minimal stress. Its adaptive core design enables it to start shaping a root canal at size 15 and to attain size 30 with 0.04 taper [23]. This was verified by fitting a gutta-percha cone size 30, 0.04 taper to within 0.5 mm of the WL. Previous studies showed that XPS was effective in preparing oval-shaped and large canals by addressing its 3-dimensional structure of the root canal space [23–25]. Additionally, XPS was able to perform acceptable shaping in canals with curvatures of 10–20° [26, 27] and 25–40° [28] without any adverse effects. In this study, XPS was able to shape severely curved canals (60°) with minimal canal transportation.

The WOG with the apical size of 0.25 mm and variable taper design of 7% maintained the canal exit in the original place with slight transportation. Previous studies revealed the ability of WOG to better perform canal shaping when compared with WaveOne [11], ProTaper Gold [14], ProTaper Next [15], and One Shape [15], respectively. Also, Özyürek et al. [12] found that the WOG caused a lower level of resin removal in simulated canals with double curvature. These results could be explained by the improved flexibility of WOG that would reduce canal transportation and maintain the canal geometry. The shaped canals demonstrated some degrees of canal transportation which is consistent with previous studies [11, 13–15]. The slight canal transportation in XPS and WOG toward the outer side at the canal exit level might be attributed to the tendency of the NiTi file to return to its original shape by the restraining forces of the file [3].

Survival of root canal-treated teeth depends not only on the quality of root canal treatment but also on the amount of remaining tooth structure [30]. Compared with XPS, the more significant removal of resin by WOG at higher levels would be due to differences in file geometry as evident previously, wherein a greater taper NiTi file induces more stress on the canal wall [31]. Bayram et al. [18] showed that XPS caused no new microcracks in the dentin which is explained by the minimal stress it induces on the canal wall [17]. Collectively, shaping the canal with XPS would preserve structural integrity more than WOG. Further studies are required to assess the impact of shaping the canals with the tested systems on stress development and microcrack formation.

Previous studies showed that XPS has excellent resistance to cyclic fatigue compared to WOG [19–22]. However, the torque at failure was found to be very low (0.84 ± 0.11 N·cm) [21] which is due to the file design. In this study, one XPS file fractured in the sixth use while none of the WOG files fractured throughout the experiment. Also, file deformation was noticed in four XPS files and three WOG files after a certain number of uses. These deformations are not only due to canal curvature but also to the narrow canal space which could develop more resistance to the apical trajectory of the file and high torque will be required to shape the canal. It is noteworthy that XPS has high angular distortion [21 which could be clinically useful as an indicator of file deformation. Therefore, the file should be inspected before each use to prevent the incidence of fracture.

The XPS file was able to shape the canal quicker than the WOG file. One of the primary factors in evaluating the efficiency of endodontic files is the shaping time of a root canal. This factor is dependent on the operator experience, the number of files used to shape a canal, the file type, and shaping technique employed. One operator, who was trained on using the tested systems, has shaped all the canals.

In the current experiment, plastic models were immersed in water bath at 35°C to let XPS file behave in its intended performance where, upon exposure to intracanal temperature, the martensitic phase of the file converts to the austenitic phase and this will expand the file in a way that will produce preparation taper of 4% throughout the canal length [18]. Besides that, using simulated intracanal temperature would mimic the clinical situation and increase the reliability of the results.

The results of the current study should be interpreted with caution due to many reasons. First, the evaluation of a three-dimensional shaped canal is restricted to two dimensions. Second, the resin block has different hardness than human dentin. Third, resin blocks demonstrated different thermal properties compared to human dentin [32]. However, for the anatomic variations that natural teeth have [33], it should be stated that the simulated resin model is considered a valid experimental tool for shaping studies with standardized taper, length, curvature, and hardness [34].

## 5. Conclusion

Within the limitation of this in vitro study on plastic models, XPS and WOG files can be used to shape severely curved canals as they showed the ability to maintain the original shape with minimal transportation. Both file systems showed signs of deformation after use with a lower number of deformed files which were observed in WOG throughout the experiment.

## Figures and Tables

**Table 1:** Canal transportation values in the tested groups at three levels. Negative and positive values mean the transportation was toward the outer and inner sides, respectively.

Level	System	Mean	Std. deviation	*P* value
0 mm	WOG	−0.0154	0.03278	>0.05
	XPS	−0.0068	0.03482	

3 mm	WOG	0.0354	0.05977	>0.05
	XPS	0.0434	0.05660	

6 mm	WOG	−0.0326	0.07617	>0.05
	XPS	−0.0346	0.09365	

## Data Availability

The datasets used and/or analyzed during the current study are available from the corresponding author on reasonable request.
